# Effects of Winter Cover Crops Residue Returning on Soil Enzyme Activities and Soil Microbial Community in Double-Cropping Rice Fields

**DOI:** 10.1371/journal.pone.0100443

**Published:** 2014-06-23

**Authors:** Tang Hai-Ming, Xiao Xiao-Ping, Tang Wen-Guang, Lin Ye-Chun, Wang Ke, Yang Guang-Li

**Affiliations:** 1 Hunan Soil and Fertilizer Institute, Changsha, PR China; 2 Guizhou Academy of Tobacco Science, Guiyang, PR China; Wageningen University, Netherlands

## Abstract

Residue management in cropping systems is useful to improve soil quality. However, the studies on the effects of residue management on the enzyme activities and microbial community of soils in South China are few. Therefore, the effects of incorporating winter cover crop residue with a double-cropping rice (*Oryza sativa* L.) system on soil enzyme activities and microbial community in Southern China fields were studied. The experiment has conducted at the experimental station of the Institute of Soil and Fertilizer Research, Hunan Academy of Agricultural Science, China since winter 2004. Four winter cropping systems were used: rice–rice–ryegrass (*Lolium multiflorum* L.) (R-R-Ry), rice–rice–Chinese milk vetch (*Astragalus sinicus* L.) (R-R-Mv), rice–rice–rape (*Brassica napus* L.) (R-R-Ra) and rice–rice with winter fallow (R-R-Fa). The result indicated that the enzyme activities in the R-R-Ry, R-R-Mv and R-R-Ra systems were significantly higher (*P*<0.05) than in the R-R-Fa system during the early and late rice season. The β-glucosidase activities reached peak values at the tillering stage after residue application, and alkaline phosphatase activities reached peak values at the booting stage after residue application, respectively, the activities of β-glucosidase and alkaline phosphatase gradually decreased after this. Arylsulfatase activities reached peak values at the maturity stage. Arylamidase activities reached peak values at the maturity stage. The numbers of aerobic bacteria, actinomycete and fungus of residue treatments were significantly higher (*P*<0.05) than that the R-R-Ra system. However, the number of anaerobic bacteria under the R-R-Ry and R-R-Mv systems was significantly lower (*P*<0.05) than that under the R-R-Fa system during early rice and late rice growth stage. Thus, incorporation of winter cover crops into rotations may increase enzyme activities and microbial community in soil and therefore improve soil quality.

## Introduction

Enzymes play an important role in the cycling of nutrients in nature, and soil enzyme activity can be used as an index of soil microbial activity and fertility [1]. Soil enzymes are involved in energy transfer, and consequently affect environmental quality and crop productivity [2], [3]. The number of enzyme production, and the activity and the stability of free and adsorbed enzymes are controlled by environmental conditions and ecological interactions. Enzymes may respond to changes in soil management more quickly than other soil variables and therefore might be useful as early indicators of biological changes. Enzyme activity profiles reflect soil functional diversity, which is influenced by the genetic diversity of soil microorganisms, plants and animals and is closely related to environmental factors and ecological interactions [4]. Among the different enzymes in soils, arylamidase, alkaline phosphatase, β-glucosidase and arylsulfatase are important for the transformation of plant nutrients. β-glucosidase catalyzes glucose formation and is an important enzyme in the terrestrial carbon cycle, glucose being an important energy source for microbial biomass [3]. Phosphatases play an important role in transforming organic phosphorus into inorganic forms that are suitable for plants. Arylamidase catalyzes one of the most important reactions in N mineralization: it releases amino acids that are used as substrates for amidohydrolases from soil organic matter. Arylsulfatase is an extracellular enzyme that catalyses the hydrolysis of organic sulphate esters, releasing SO_2_ that can be used by plants.

Soil microbes play an important role in ecosystem function and may act as filters or valves that regulate the intra-system cycling of soil nutrients [5]. Soil microbes play an important role in the maintenance of soil fertility (i.e. nutrient cycling and organic matter decomposition). The ability of microbes to maintain soil fertility and regulate nutrient cycling may be largely dependent on the composition of soil microorganisms communities [6], [7], and changes in microbial diversity or community structure could have dramatic impacts on ecosystem processes [8].

Winter cover crops, which are grown during an otherwise fallow period, are a possible means of improving nutrient dynamics in the surface layer of intensively managed cropping systems. Hermawan and Bomke [9] suggested that growing winter cover crops such as annual ryegrass may protect aggregate breakdown during winter and result in a better soil structure after spring tillage, as opposed to leaving soil bare. Other potential benefits of winter cover crops are the prevention of nitrate leaching [10]; weed infestation [11]; and improvement of soil water retention, soil organic matter content and microbial activity [12]. Recycling of crop residues has been suggested to improve overall soil conditions, reduce the requirement for N fertilizers and support sustainable rice (*Oryza sativa* L.) productivity. Chinese milk vetch (*Astragalus sinicus* L.), ryegrass (*Lolium multiflorum* L.) and rape (*Brassica napus* L.) are the main winter cover crops in China.

In recent years, many studies have shown that the enzyme and microbial activities of soil are affected by soil tillage and residue management [13], application of fertilizer and organic matter [14], [15], crop rotations [16], and field management [17], [18]. However, relatively few studies on soil enzyme activities and soil microbes in double-rice cropping system with winter cover crop rotations have been conducted in southern China.

Our relatively rare study was conducted in a double-cropping paddy field, the winter cover crops tested were ryegrass (*Lolium multiflorum* L.), Chinese milk vetch (*Astragalus sinicus* L.), and rape (*Brassica napus* L.), and a control plot with weed and no cover crop was also used. Usually, enzymes and microbial activities affected by management practices were used as indicators of ecological stability. The objectives of this research were to determine the effects of different winter cover crops residues returning on soil enzyme activities and soil microbial community in a double-cropping rice system.

## Materials and Methods

### Sites

**Figure 1:**
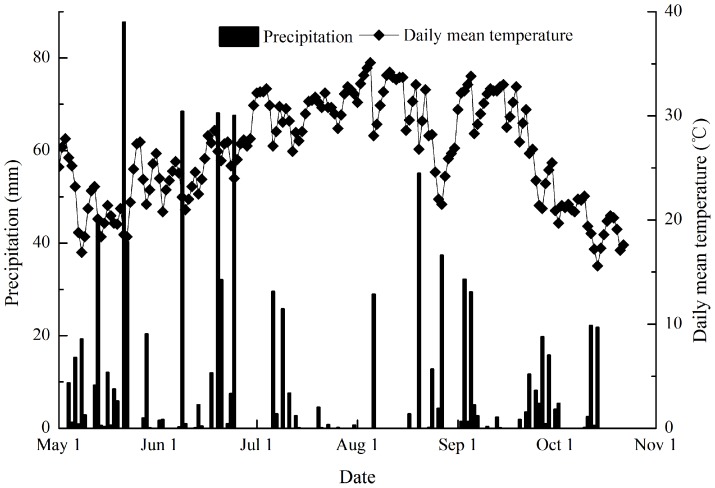
Daily precipitation and daily mean temperature at the study site during the experimental period.

The experiment has conducted at the experimental station of the Institute of Soil and Fertilizer Research, Hunan Academy of Agricultural Science, China (28°11′58″ N, 113°04′47″ E) since winter 2004. The typical cropping system in this area is double-cropping rice. The soil type is a Fe–accumuli–Stagnic Anthrosol derived from Quaternary red clay (clay loam). The characteristics of the surface soil (0–20 cm) are: pH 5.40, soil organic carbon (SOC) 13.30 g kg^−1^, total nitrogen 1.46 g kg^−1^, available N 154.5 mg kg^−1^, total phosphorous 0.81 g kg^−1^, available P 39.2 mg kg^−1^, total potassium 13.0 g kg^−1^, and available potassium 57.0 mg kg^−1^ [19]. All these values were tested before the experiment in 2004. This region has the subtropical monsoonal humid climate, with a long hot period and short cold period. The average annual precipitation is approximately 1500 mm and the annual mean temperature is 17.1°C, and the annual frost-free period is approximately from 270 days to 310 days. The daily precipitation and daily mean temperature data during the 2010 early and late rice growing season is presented in Fig. 1.

### Experimental design and soil sampling

The cropping system was the winter cover crops were planted after double cropping rice harvested every year. The study was continuously conducted for 7 years after straw recycling of the winter cover crop. The four cropping systems used were rice–rice–ryegrass (R-R-Ry), rice–rice–Chinese milk vetch (R-R-Mv), rice–rice–rape (R-R-Ra) and rice–rice with winter fallow (R-R-Fa). A randomized block design was adopted in plots, with three replications. The plot area was 1.1 m^2^ (1 m×1.1 m). Before sowing the winter cover crops, 75.0 kg ha^−1^ N (163.0 kg urea) and 45.0 kg ha^−1^ P_2_O_5_ (375.0 kg diammonium phosphate) were applied as basal fertilizer. The winter cover crops planted in winter 2004 were cut and incorporated into soil by means of a disk plow (conventional tillage) in the following spring. When winter cover crops were harvested, the straw residue of ryegrass, Chinese milk vetch, and rape straw residue were weighted and returned at the same quantity of 22500.0 kg ha^−1^ to the soil surface. And the plots were plowed once to a depth of 20 cm using a moldboard plow on the fifteen day before rice seedling transplanting.

One-month-old seedlings were transplanted at a density of 150,000 plants ha^−1^ (one seed per 16 cm×16 cm), with 2–3 plants per hill. For both early and late rice, basal fertilizer was applied at the rate of 150.0 N kg ha^−1^ as urea (60%, basal; 40%, top-dressed at the tillering stage), 75.0 kg ha^−1^ P_2_O_5_ as diammonium phosphate and 120.0 kg ha^−1^ K_2_O as potassium sulfate.

Data were collected during the early and late rice growth period in 2010. Soil samples in the ploughed layer (0–20 cm) were collected from the centre of four hills of rice plants by using a drill at different rice growth periods.

### Laboratory analyses

The soil samples were passed through a 2-mm sieve and kept moist in a refrigerator at 4°C until use. Arylamidase (EC 3.4.11.2) activity was assayed by incubating 1.0 g moist soil with 3.0 mL of 0.1 M THAM buffer (pH 8.0) and 1.0 mL of an 8.0 mM solution of l-leucine β-naphthylamide hydrochloride [20]. Alkaline phosphatase (EC 3.1.3.1), β-glucosidase (EC 3.2.1.21) and arylsulfatase (EC 3.1.6.1) activities were determined as described by Tabatabai [3], and the activity was reported as µg *p*-nitrophenol g^−1^ h^−1^. All the measurements were repeated for three times.

Colony forming units (CFUs) of soil anaerobic bacteria, aerobic bacteria, actinomycetes, and fungi were enumerated by a 10-fold dilution plate technique. And the number of aerobic bacteria were identified by spreading 100 µl of diluted sample on LB agar medium. The medium for actinomycetes contained 1% (w/v) soluble starch, 0.2% (w/v) (NH_4_)_2_SO_4_, 0.1% (w/v) K_2_HPO_4_, 0.1% (w/v) MgSO_4_·7H_2_O, 0.7% (w/v) NaCl, 0.3% (w/v) CaCO_3_, and 2% (w/v) agar. The number of CFUs of actinomycetes formed on this medium was determined by probing colonies that developed with a dissecting needle: if the colony remained as a discrete, small mass, it was considered to be an actinomycete. The number of CFUs of fungi was estimated on Martin's agar medium containing 1.25 g streptomycin l^−1^ and 33 mg rose bengal l^−l^. Three replicates of the inoculated agar plates were incubated at 28°C for 3 d for bacteria, 5 d for fungi, and 7 d for actinomycetes, after which colonies were counted [17]. The number of aerobic bacteria was determined according to the method described by Oliveira et al. [21]. The number of anaerobic bacteria was determined according to the method described by Min et al. [22].

### Statistical analysis

All data were expressed as mean ± standard error. The data were analyzed as a randomized complete block, using the PROC ANOVA procedure of SAS [23]. Mean values were compared using the least significant difference (LSD) test, and a probability value of 0.05 was considered to indicate statistical significance.

## Results

### Dynamics of β-glucosidase activity during the rice growth period

**Figure 2:**
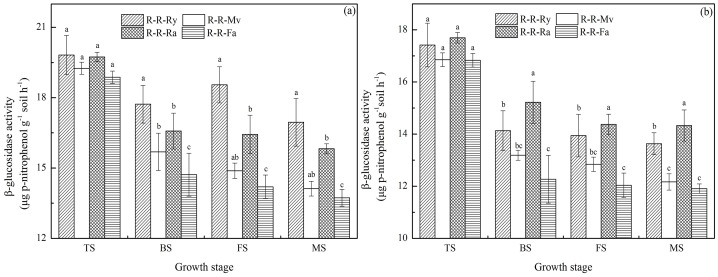
Dynamics of β-glucosidase activity in paddy fields during the rice growth period (a for the early rice season and b for the late rice season). R-R-Ry: rice–rice–ryegrass cropping system; R-R-Mv: rice–rice–Chinese milk vetch cropping system; R-R-Ra: rice–rice–rape cropping system; R-R-Fa: rice–rice cropping system with winter fallow. TS: tillering stage; BS: booting stage; FS: full heading stage; MS: maturity stage. Error bars represent the standard error of mean. Different letters indicate significance at *P*<0.05, according to the least significant difference test.

In the early rice season, the activity of β-glucosidase in soils was significantly affected by the residue management practice. The R-R-Ry had the highest β-glucosidase activity and the R-R-Fa had the lowest activity (Fig. 2), and the activity decreased in the following order: R-R-Ry>R-R-Ra≈R-R-Mv>R-R-Fa. In the late rice season, the highest activity was the treatment that the rape was used as residues, and the activity trend was as follows: R-R-Ra>R-R-Ry≈R-R-Mv>R-R-Fa.

### Dynamics of alkaline phosphatase activity during the rice growth period

**Figure 3:**
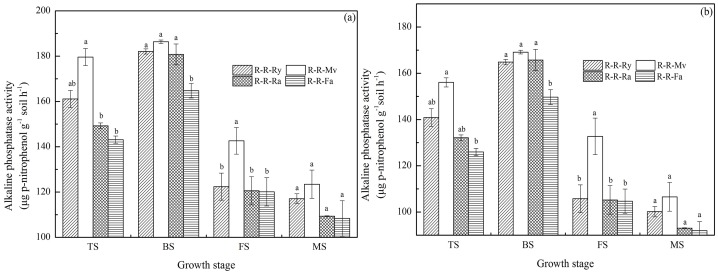
Dynamics of alkaline phosphatase activity in paddy fields during the rice growth period (a for the early rice season and b for the late rice season). R-R-Ry: rice–rice–ryegrass cropping system; R-R-Mv: rice–rice–Chinese milk vetch cropping system; R-R-Ra: rice–rice–rape cropping system; R-R-Fa: rice–rice cropping system with winter fallow. TS: tillering stage; BS: booting stage; FS: full heading stage; MS: maturity stage. Error bars represent the standard error of mean. Different letters indicate significance at *P*<0.05, according to the least significant difference test.

With the addition of the winter cover crop residues, alkaline phosphatase activity in the R-R-Ry, R-R-Mv, and R-R-Ra soils was higher than that in R-R-Fa (Fig. 3). In other words, the alkaline phosphatase activity was enhanced by the application of cover crop residues in the rice growth season. The alkaline phosphatase activity of soils in the early rice season was higher than that in the late rice season. In the early and late rice seasons, alkaline phosphatase activity decreased as follows: R-R-Mv>R-R-Fa, but there were no significant differences (*P*>0.05) among other treatments. And there were no significant differences (*P*>0.05) in alkaline phosphatase activity under the R-R-Ry, R-R-Mv, R-R-Ra and R-R-Fa systems at the maturity stages. In the early and late rice seasons, the alkaline phosphatase activity of different soils collected at different growth stages was in the range of 108.34–186.37 and 92.02–169.12 µg *p*-nitrophenol g^−1^ soil h^−1^, respectively. The highest activities were detected at the booting stage.

### Dynamics of arylsulfatase activity during the rice growth period

**Figure 4:**
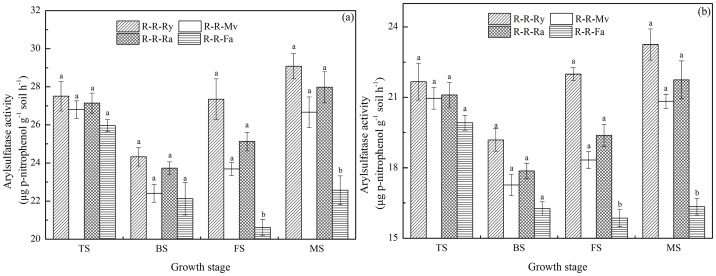
Dynamics of arylsulfatase activity during the rice growth period (a for the early rice season and b for the late rice season). R-R-Ry: rice–rice–ryegrass cropping system; R-R-Mv: rice–rice–Chinese milk vetch cropping system; R-R-Ra: rice–rice–rape cropping system; R-R-Fa: rice–rice cropping system with winter fallow. TS: tillering stage; BS: booting stage; FS: full heading stage; MS: maturity stage. Error bars represent the standard error of mean. Different letters indicate significance at *P*<0.05, according to the least significant difference test.

In the early and late rice seasons, arylsulfatase activity in the soils was in the range of 20.61–29.08 and 15.86–23.25 µg *p*–nitrophenol g^−1^ soil h^−1^, respectively (Fig. 4). At the tillering and booting stage, there was no significant difference in arylsulfatase activity among the R-R-Ry, R-R-Mv, R-R-Ra and R-R-Fa, but the activity of this enzyme in R-R-Ry, R-R-Mv, and R-R-Ra soils was significantly (*P*<0.05) higher than that in R-R-Fa at the full heading and maturity stage.

### Dynamics of arylamidase activity during the rice growth period

**Figure 5:**
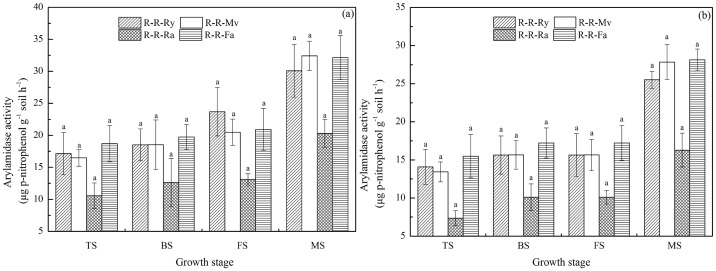
Dynamics of arylamidase activity during the rice growth period (a for the early rice season and b for the late rice season). R-R-Ry: rice–rice–ryegrass cropping system; R-R-Mv: rice–rice–Chinese milk vetch cropping system; R-R-Ra: rice–rice–rape cropping system; R-R-Fa: rice–rice cropping system with winter fallow. TS: tillering stage; BS: booting stage; FS: full heading stage; MS: maturity stage. Error bars represent the standard error of mean. Different letters indicate significance at *P*<0.05, according to the least significant difference test.

Straw recycling of winter cover crops did not significantly affect arylamidase activity in the soil (Fig. 5). At 0–20 cm soil depth, there was no significant difference (*P*>0.05) in arylamidase activity under the R-R-Ry, R-R-Mv, R-R-Ra and R-R-Fa. In the early and late rice seasons, arylamidase activity changed in the range of 10.55–32.42 and 7.34–28.14 µg *p*-nitrophenol g^−1^ soil h^−1^, respectively.

### Enumeration of aerobic bacteria, anaerobic bacteria, actinomycetes and fungi in the paddy soil

Significant differences (*P*<0.05) were observed in the numbers of culturable aerobic bacteria, anaerobic bacteria, actinomycetes, and fungi between samplings throughout the rice growth season. The number of aerobic bacteria decreased in the order R-R-Ry>R-R-Mv>R-R-Ra>R-R-Fa in the early rice season and in the order R-R-Ra>R-R-Mv>R-R-Ry>R-R-Fa in the late rice season (Table 1). The number of anaerobic bacteria in the paddy soil under the R-R-Ry and R-R-Mv systems was significantly lower than that in the R-R-Ra system, while there was no significant difference between R-R-Ra and R-R-Fa. However, the numbers of actinomycetes and fungi in the soil with straw recycling of winter cover crops were significantly higher (*P*<0.05) than that in the winter fallow soil throughout the main growth stages. And the number of fungi decreased in the order R-R-Ra>R-R-Ry>R-R-Mv>R-R-Fa in the early rice season and in the order R-R-Mv>R-R-Ry≈R-R-Ra>R-R-Fa in the late rice season. And the number of actinomycete decreased in the order R-R-Mv>R-R-Ry≈R-R-Ra>R-R-Fa in the early and late rice season.

**Table 1:** Variations in the number (10^4^ CFUs g^−1^ dry soil) of aerobic bacteria, anaerobic bacteria, actinomycetes and fungi in a paddy soil under different cropping systems.

Items	Treatment	Early rice				Late rice			
		TS	BS	CS	MS	TS	BS	CS	MS
Aerobic bacteria	R-R-Ry	618.33±5.12a	453.47±7.00a	359.37±4.93a	173.07±5.96c	343.73±5.41c	219.13±5.32c	130.93±2.49c	58.33±1.06c
	R-R-Mv	566.03±4.76b	408.67±8.80b	345.03±3.39b	487.27±2.93b	461.83±4.76b	319.13±4.76b	225.63±3.18b	107.03±1.85b
	R-R-Ra	337.53±5.35c	212.60±3.74c	150.70±4.07c	751.87±5.41a	550.47±5.12a	394.37±5.42a	295.97±3.54a	127.37±2.05a
	R-R-Fa	258.63±6.09d	138.23±1.55d	98.60±1.79d	61.43±4.43d	211.93±6.09d	122.63±4.57d	62.43±1.67d	38.13±1.12d
Anaerobic bacteria	R-R-Ry	29.30±0.78b	23.80±0.71b	21.57±0.57c	27.33±0.61a	24.29±0.74b	19.42±0.72c	17.36±0.62c	17.19±0.62a
	R-R-Mv	31.93±0.55b	26.53±0.72b	23.07±0.58bc	18.07±0.43c	26.77±0.51b	22.33±0.88b	18.88±0.52bc	14.25±0.59c
	R-R-Ra	41.67±0.70a	35.83±0.76a	28.57±0.43a	24.93±0.82c	36.50±0.72a	31.50±0.71a	23.80±0.43a	18.11±0.59c
	R-R-Fa	39.80±1.96a	33.77±1.11a	24.23±0.58b	23.07±0.44b	34.79±1.96a	29.02±1.05a	19.60±0.56b	18.01±0.48b
Fungi	R-R-Ry	2.87±0.05a	2.03±0.03b	1.62±0.04b	0.65±0.03b	2.53±0.05b	1.75±0.04b	1.29±0.04b	0.40±0.03b
	R-R-Mv	2.74±0.03b	1.90±0.05c	1.42±0.03c	0.59±0.04b	2.42±0.03b	1.66±0.05b	1.06±0.02c	0.36±0.04b
	R-R-Ra	2.99±0.05a	2.44±0.02a	2.11±0.05a	1.09±0.03a	2.72±0.04a	2.11±0.02a	1.74±0.06a	0.81±0.03a
	R-R-Fa	2.20±0.02c	1.55±0.02d	1.34±0.03c	0.27±0.04c	1.94±0.02c	1.31±0.03c	0.99±0.04c	0.19±0.04c
Actinomycetes	R-R-Ry	9.07±0.29b	9.47±0.25b	9.93±0.43b	17.47±0.35a	6.93±0.28b	6.94±0.23b	7.30±0.46b	14.32±0.35a
	R-R-Mv	10.27±0.26a	11.50±0.32a	12.87±0.44a	18.47±0.35a	8.10±0.26a	8.92±0.32a	10.16±0.44a	15.23±0.35a
	R-R-Ra	8.10±0.21bc	8.73±0.38bc	9.17±0.23bc	12.77±0.43b	5.93±0.20c	6.53±0.41bc	6.40±0.23bc	9.73±0.43b
	R-R-Fa	7.13±0.41c	7.87±0.43c	8.50±0.36c	10.20±0.26c	4.99±0.41c	5.62±0.43c	5.87±0.36c	7.05±0.26c

R-R-Ry: rice–rice–ryegrass cropping system; R-R-Mv: rice–rice–Chinese milk vetch cropping system; R-R-Ra: rice–rice–rape cropping system; R-R-Fa: rice–rice cropping system with winter fallow.

TS: tillering stage; BS: booting stage; FS: full heading stage; MS: maturity stage.

Values are presented as mean ± SE (n = 3). Means in each column with different letters are significantly different at the *P*<0.05 level.

## Discussion

### Soil quality and winter cover crops residue applied

Residue management in cropping systems is believed to improve the quality of soil [7]. In our study, adding winter cover crops and returning the straws to the field is benefit to increase the soil quality and rice yield in double-cropping rice. And it is beneficial in resources efficiency increase. Soil enzyme activities and soil microbial community play an important role in ecosystem function and cycling of soil nutrients. And the ability of maintain soil fertility or regulate nutrient cycling may be largely dependent on the composition of soil microbial communities and soil enzyme activities [7]. In our study, the activities of all four enzymes with winter cover crop residue were generally higher than the fallow fields because the increases of soil enzyme activity due to add organic matter to soils, this is same with other people's studies [4], [24]. In addition, we were able to differentiate temporal variations in soil enzyme activities from changes in the enzyme activity in response to winter cover crop residue decomposition, different organic inputs (winter cover crop residue), straw incorporation or crop rotation.

Management practices (e.g. crop rotation, mulching, tillage and application of fertilizers and organic matter) may have diverse effects on the numbers of microorganisms in soil [14], [15], [17]. Thus, incorporation of winter cover crops into rotations may increase microbial community in soil compared to the R-R-Fa system. This might have been because there was more decomposable organic material in the adding winter cover crops soil which favored soil microbe. And the numbers of microorganisms were improved after returning straws of winter cover crops into the soil due to the supplements of carbon source. Soil microorganisms are mostly heterotrophic and use organic carbon as carbon and energy sources.

### Soil enzyme activities, microbial community and winter cover crops residue applied

β-glucosidase is the rate-limiting enzyme in the microbial degradation of cellulose to glucose and plays a crucial role in the C cycle in soils. We found a significant difference in β-glucosidase activity between the R-R-Ry, R-R-Mv and R-R-Ra treatments at the rice growth stages, which is probably related to the different C inputs by the different winter cover crops [25]. In the early and late rice seasons, alkaline phosphatase activity decreased as follows: R-R-Mv>R-R-Fa, but there were no significant differences (*P*>0.05) between other treatments. There was no significant difference (*P*>0.05) in arylamidase activity under the R-R-Ry, R-R-Mv, R-R-Ra and R-R-Fa systems in the early and late rice seasons. Also, other investigators have reported that Chinese milk vetch residue contains enzymes (phosphatase) [26]. The different ranking of treatments in soil enzyme activities might be related to the kinds of winter cover crops straw type. This might have been because there was significant difference decomposable organic material in the winter cover crops straw-returned soil which favored soil enzyme activities [27], [28]. Arylsulfatase is produced by both plants and microorganisms [15]. It is an extracellular enzyme that catalyses the hydrolysis of organic sulphate esters, releasing SO_4_ that can be used by plants. In our study, the activity decreased in the following order: R-R-Ry>R-R-Ra>R-R-Mv in the early and late rice season. These observations indicate that ryegrass and rape made a higher contribution to the active pool of C in soil organic matter than Chinese milk vetch, which may explain the higher immobilization in rhizosphere soil containing ryegrass and rape residue than in rhizosphere soil containing Chinese milk vetch residue. Thus, there may be qualities unique to each cover crop that increases the activity of specific enzymes [28].

Yoshinari et al. [29] observed that rice straw affected soil microbial community structure. In the laboratory, we verified the increased numbers of bacteria, actinomycetes, and fungi in the soil with winter cover crop residue. In our study, the number of aerobic bacteria decreased in the order R-R-Ry>R-R-Mv>R-R-Ra>R-R-Fa in the early rice season and in the order R-R-Ra>R-R-Mv>R-R-Ry>R-R-Fa in the late rice season. This might have been because there was significant difference of decay rates in the winter cover crops straws-returned soil which favored aerobic bacteria. And the numbers of aerobic bacteria were increased under R-R-Ry and R-R-Ra systems in the early and late rice season, respectively. The numbers of anaerobic bacteria in the paddy soil under the R-R-Ry and R-R-Mv systems were significantly lower than that in the R-R-Ra system. The reason for this difference is being investigated. On the one hand, both of the soils in which rape straws had been incorporated showed considerable increases in the numbers of culturable anaerobic microorganisms compared to the ryegrass and Chinese milk vetch straws-added soil (Table 1). On the other hand, this might have been because there was more decomposable organic material in the rape straw-added soil resulting in more consumption of O_2_ which favored anaerobes [17]. The limitations of cultivation based assessment of anaerobic microorganisms should be further studied.

Numerous studies indicate that fungi play an important role in both the formation and stabilization of soil aggregates but they are sensitive to disturbance, pollution and environmental change [30], [31]. In this study, the number of fungi decreased in the order R-R-Ra>R-R-Ry>R-R-Mv in the early rice season and in the order R-R-Mv>R-R-Ry≈R-R-Ra in the late rice season. This may be that the changes of soil environmental on fungal diversity could influence ecosystem function via decomposition of different winter cover crops straws, fungi populations increased by returning rape and Chinese milk vetch straws in the early and late rice season, respectively. But the limitations of cultivation based assessment of fungi should be further studied. Furthermore, the number of actinomycete decreased in the order R-R-Mv>R-R-Ry≈R-R-Ra>R-R-Fa in the early and late rice season. Different ranking of treatments in the number of actinomycetes might be related to the decomposition rates of winter crop species [32]. In this study, it was believed that the use of Chinese milk vetch residue as organic manure resulted in an increase in the number of actinomycete. In general, the differences in the composition of the straws may have caused differences in the microbial and which resulted in the significant differences in the numbers of bacteria, actinomycetes, and fungi between the paddy soils added with ryegrass, Chinese milk vetch and rape straws. In our study, the approaches for enumeration of microbial groups are severely limited by the fact that only a fraction of microorganisms is amenable to cultivation using the traditional methods applied here. For example, it doesn't allow all aerobic microorganisms to grow by using the LB agar medium. Therefore, some advanced methods to study the aerobic microorganisms should be investigated. Further studies could be helpful to better understand on how these changes in the number of microbial might actually impact microbial functions and nutrient availability in paddy soils.

## Conclusions

This study revealed that the soil enzyme activities and soil microbes were affected by residue management practices during the early and late rice growth stages, and that these two factors could be used as potential soil quality indicators. Application of winter cover crop residue can improve soil β-glucosidase, alkaline phosphatase and arylsulfatase activities. The activity of β-glucosidase in soils under R-R-Ry was increased 0.95, 3.01, 4.35, 3.22 µg *p*-nitrophenol g^−1^ soil h^−1^ in the early rice season, the activity of β-glucosidase in soils under R-R-Ra was increased 0.86, 2.95, 2.34, 2.42 µg *p*-nitrophenol g^−1^ soil h^−1^ compared with the R-R-Fa in the late rice season. Moreover, the activity of alkaline phosphatase under R-R-Mv was increased 36.50, 21.62, 22.53, 15.13 µg *p*-nitrophenol g^−1^ soil h^−1^ and 30.06, 19.43, 27.99, 14.58 µg *p*-nitrophenol g^−1^ soil h^−1^ in the early and late rice season, respectively. The activity of arylsulfatase in R-R-Ry was increased 1.55, 2.20, 6.74, 6.51 µg *p*-nitrophenol g^−1^ soil h^−1^ and 1.75, 2.93, 6.13, 6.91 µg *p*-nitrophenol g^−1^ soil h^−1^ in the early and late rice season, respectively. In contrast, no significant effect on arylamidase activity was found. The population of soil microbes also increased when winter cover crop residue was applied. The number of aerobic bacteria under the R-R-Ry systems was increased 359.70, 315.24, 260.77, 111.64×10^4^ CFUs g^−1^ dry soil and 131.80, 96.50, 68.50, 20.20×10^4^ CFUs g^−1^ dry soil compared with the R-R-Fa during early and late rice growth stage. The number of anaerobic bacteria under the R-R-Ra systems was increased 1.87, 2.06, 4.34, 1.86×10^4^ CFUs g^−1^ dry soil and 1.71, 2.48, 4.20, 0.10×10^4^ CFUs g^−1^ dry soil during early and late rice growth stage. The number of actinomycetes bacteria under the R-R-Mv systems was increased 3.14, 3.63, 4.37, 8.27×10^4^ CFUs g^−1^ dry soil and 3.11, 3.30, 4.29, 8.18×10^4^ CFUs g^−1^ dry soil during early and late rice growth stage. Thus, the results indicated that the management practices of winter cover crop residue returning with rotations can improve soil enzyme activities and the soil microbial community.

## References

[1] Benitez E, Melgar R, Sainz H, Gomez M, Nogales R (2000) Enzyme activities in the rhizosphere of pepper (CapsicumannuumL.) grown with olive cake mulches. Soil Biology & Biochemistry 32: 1829–1835.

[2] Dick RP (1994) Soil Enzyme Activities as Indicators of Soil Quality. In: Doran JW, Coleman DC, Bezdicek DF, Stewart BA (Eds.). Defining soil quality for a sustainable environment. American Society of Agronomy, Madison, WI, 107–124.

[3] Tabatabai MA (1994) Soil Enzymes. In: Page AL, Miller RH, Keeney DR. (Eds.). Methods of soil analysis. American Society of Agronomy, Madison, 775–833.

[4] Nannipieri P, Kandeler E, Ruggiero P (2002) Enzyme Activities and Microbiological and Biochemical Processes in Soil. In: Burns RG, Dick RP. (Eds.). Enzymes in the environment. Activity, Ecology and Applications. Dekker, New York, 1–33.

[5] Pastor J, Aber JD, McClaugherty CA, Melillo JM (1984) Above–ground production and N and P cycling along a nitrogen mineralization gradient on Blackhawk Island, Wisconsin. Ecology 65: 256–268.

[6] Robertson GP, Paul EA, Harwood RR (2000) Greenhouse gases in intensive agriculture: contributions of individual gases to the radiative forcing of the atmosphere. Science 289: 1922–1925. 10988070 10.1126/science.289.5486.1922

[7] Singh BK, Bardgett RD, Smith P, Reay DS (2010) Microorganisms and climate change: terrestrial feedbacks and mitigation options. Nature Reviews Microbiology 8: 779–790. 20948551 10.1038/nrmicro2439

[8] Prosser JI (2002) Molecular and functional diversity in soil micro–organisms. Plant and Soil 244: 9–17.

[9] Hermawan B, Bomke AA (1997) Effects of winter cover crops and successive spring tillage on soil aggregation. Soil & Tillage Research 44: 109–120.

[10] McCracken DV, Smith MS, Grove JH, MacKown CT, Blevins RL (1994) Nitrate leaching as influenced by cover cropping and nitrogen source. Soil Science Society of America Journal 58: 1476–1483.

[11] Barnes JP, Putnam AR (1983) Rye residues contribute weed suppression in no–tillage cropping systems. Journal of Chemical Ecology 9: 1045–1057. 24407799 10.1007/BF00982210

[12] Powlson DS, Prookes PC, Christensen BT (1987) Measurement of soil microbial biomass provides an early indication of changes in total soil organic matter due to straw incorporation. Soil Biology & Biochemistry 19(2): 159–164.

[13] Ekenler M, Tabatabai MA (2003) Tillage and residue management effects on β–glucosaminidase activity in soils. Soil Biology & Biochemistry 35: 871–874.

[14] Carmine C, Magda C, Maria DRP, Patrizia R, Pacifico R (2004) Effects of municipal solid waste compost amendments on soil enzyme activities and bacterial genetic diversity. Soil Biology & Biochemistry 36: 1595–1605.

[15] Tejada M, Garcia C, Gonzalez JL, Hernandez MT (2006) Use of organic amendment as a strategy for saline soil remediation: Influence on the physical, chemical and biological properties of soil. Soil Biology & Biochemistry 38: 1413–1421.

[16] Hamido SA, Kpomblekou AK (2009) Cover crop and tillage effects on soil enzyme activities following tomato. Soil & Tillage Research 105: 269–274.

[17] Wu WX, Ye QF, Min H, Duan XJ, Jin WM (2004) Bt–transgenic rice straw affects the culturable microbiota and dehydrogenase and phosphatase activities in a flooded paddy soil. Soil Biology & Biochemistry 36: 289–295.

[18] Anna KB, Richard PD (1999) Field management effects on soil enzyme activities. Soil Biology and Biochemistry 31: 1471–1479.

[19] Klose S, Koch J, Baucker E, Makeschin F (2001) Indicative properties of fly-ash affected forest soils in Northeastern Germany. Journal of Plant Nutrition and Soil Science 164: 561–568.

[20] Acosta–Marti'nez V, Tabatabai MA (2000) Arylamidase activity of soils. Soil Science Society of America Journal 64: 215–221.

[21] Oliveira AP, Pampulha ME, Bennett JP (2008) A two-year field study with transgenic *Bacillus thuringiensis* maize: Effects on soil microorganisms. Science of the Total Environment 405: 351–357. 18656246 10.1016/j.scitotenv.2008.05.046

[22] Min H, Ye YF, Chen ZY, Wu WX, Du YF (2001) Effects of butachlor on microbial populations and enzyme activities in paddy soil. Journal of Environmental Science and Health B 36: 581–595. 10.1081/PFC-100106187 11599722

[23] SAS Institute (2003) SAS Version 9.1.2 2002–2003. SAS Institute Inc., Cary, NC.

[24] Crecchio C, Curci M, Mininni R, Ricciuti P, Ruggiero P (2001) Short–term effects of municipal solid waste compost amendments on soil carbon nitrogen content, some enzyme activities and genetic diversity. Biology and Fertility of Soils 34: 311–318.

[25] Huang C, Deng LJ, Gao XS, Zhang SR, Luo T, et al (2010) Effects of fungal residues return on soil enzymatic activities and fertility dynamics in a paddy soil under a rice-wheat rotation in Chengdu Plain. Soil & Tillage Research 108: 16–23.

[26] Nannipieri P, Muccini L, Ciardi C (1983) Microbial biomass and enzyme activities: production and persistence. Soil Biology & Biochemistry 15: 679–685.

[27] Luo YJ, Wang ZF, Gao M, Wei CF (2011) Effects of conservation tillage on organic carbon, nitrogen and enzyme activities in a hydragric anthrosol of Chongqing, China. Energy Procedia 5: 30–36.

[28] Sharma RK, Arora DS (2011) Solid state degradation of paddy straw by *Phlebia floridensis* in the presence of different supplements for improving its nutritive status. International Biodeterioration & Biodegradation 65: 990–996.

[29] Yoshinari E, Seigo O, Hiroshi Y, Yoshio N (2001) Influence of earthworm activity and rice straw application on the soil microbial community structure analyzed by PLFA pattern. European Journal of Soil Biology 37: 269–272.

[30] David JB, Kurt AS, Juan CL, Jared LD (2012) Soil fungi influence the distribution of microbial functional groups that mediate forest greenhouse gas emissions. Soil Biology & Biochemistry 53: 112–119.

[31] Li YJ, Chen X, Shamsi IH, Fang P, Lin XY (2012) Effects of irrigation patterns and nitrogen fertilization on rice yield and microbial community structure in paddy soil. Pedosphere 22(5): 661–672.

[32] Zhang QC, Shamsi IH, Xua DT, Wang GH, Lin XY, et al (2012) Chemical fertilizer and organic manure inputs in soil exhibit a vice versa pattern of microbial community structure. Applied Soil Ecology 57: 1–8.

